# Health Risks of Hypovitaminosis D: A Review of New Molecular Insights

**DOI:** 10.3390/ijms19030892

**Published:** 2018-03-17

**Authors:** Daniela Caccamo, Sergio Ricca, Monica Currò, Riccardo Ientile

**Affiliations:** Department of Biomedical Sciences, Dental Sciences, and Morpho-functional Imaging, University of Messina, 98125 Messina, Italy; ricca.sergio85@gmail.com (S.R.); monica.curro@unime.it (M.C.); ientile@unime.it (R.I.)

**Keywords:** vitamin D, VDR, cardiovascular diseases (CVD), central nervous system (CNS), allergy, microbioma

## Abstract

Hypovitaminosis D has become a pandemic, being observed in all ethnicities and age groups worldwide. Environmental factors, such as increased air pollution and reduced ultraviolet B (UVB) irradiation, as well as lifestyle factors, i.e., decreased outdoor activities and/or poor intake of vitamin D-rich food, are likely involved in the etiology of a dramatic reduction of vitamin D circulating levels. The insufficiency/deficiency of vitamin D has long been known for its association with osteoporosis and rickets. However, in the last few decades it has become a serious public health concern since it has been shown to be independently associated with various chronic pathological conditions such as cancer, coronary heart disease, neurological diseases, type II diabetes, autoimmune diseases, depression, with various inflammatory disorders, and with increased risk for all-cause mortality in the general population. Prevention strategies for these disorders have recently involved supplementation with either vitamin D2 or vitamin D3 or their analogs at required daily doses and tolerable upper-limit levels. This review will focus on the emerging evidence about non-classical biological functions of vitamin D in various disorders.

## 1. Introduction

Recent epidemiological studies have demonstrated that an insufficiency of vitamin D (<72.5 nmol/L; <30 ng/ml), affects 50% of the population worldwide, while 1 billion people show vitamin D deficiency (<25 nmol/L; <10 ng/ml), as per the cutoffs established by the Endocrine Society Clinical Practice Guidelines [1,2,3]. Notably, hypovitaminosis D has been observed in all ethnicities and age groups, and found to be more severe in men than in women and the elderly The etiology of this pandemic includes lifestyle factors, i.e., low dietary intake and decreased outdoor activities, and environmental factors, i.e., increased air pollution, these latter both reducing the exposure to sunlight needed for UVB-mediated activation of vitamin D synthesis starting from 7-dehydrocholesterol in the epidermis [1,2,3].

The insufficiency/deficiency of vitamin D raises public health concern since it has been shown to be independently associated with a higher risk of all-cause mortality [4,5]. In fact, hypovitaminosis D has long been known to increase the risk for osteoporosis and rickets, and only in the last decades it has been linked with various chronic pathological conditions, i.e., cancer, coronary heart disease (CHD), non-insulin dependent diabetes, neurological disorders, as well as autoimmune and inflammatory diseases [5,6].

This review will focus on the molecular mechanisms involved in the non-classical roles of vitamin D in various disease paradigms.

## 2. Vitamin D Analogs

To date, five natural analogs and four synthetic analogs of vitamin D, generally classified as secosteroids, are known.

Vitamin D active as a hormone in the human body is obtained from dietary intake of its natural precursors cholecalciferol and ergocalciferol, also known as vitamin D3 and vitamin D2, having animal and vegetable origin, respectively. Vitamin D3 can also be obtained by UVB-activated photochemical conversion of pro-vitamin D3 (7-dehydrocholesterol) to pre-vitamin D3 (cholecalciferol) in the skin. Bound to vitamin D binding protein (DBP), pre-vitamin D3 is transported to the liver where it is converted into 25-hydroxyvitamin D upon 25-hydroxylase (CYP2R1)-mediated hydroxylation. A further 1-alpha-hydroxylase (CYP27B1)-mediated hydroxylation step, occurring in the kidney, is required to obtain the active form of vitamin D, 1,25-dihydroxyvitamin D, also known as calcitriol [7]. Other natural analogs of vitamin D are represented by vitamin D1 (ergocalciferol:lumisterol 1:1), vitamin D4 (22-dihydroergocalciferol) and vitamin D5 (sitocalciferol).

There are thousands of synthetic vitamin D analogs, having a wide range of therapeutic applications [7,8,9], and these include: 22-oxacalcitriol (OCT), having a wider pleiotropic action than 1,25-dihydroxyvitamin D3; falecalcitriol, that is more active than calcitriol due to a slower metabolism; two calcitriol-derived molecules, calcipotriol and paricalcitol, this latter having a side chain represented by vitamin D_2_; dihydrotachysterol (DHT), activated in the liver without need for further hydroxylation in the kidney; doxercalciferol, a pro-drug undergoing in vivo activation that displays less toxic effects than 1α-hydroxyvitamin D3 when chronically administered; and tacalcitol, derived from vitamin D_3_ [8].

**Figure 1:**
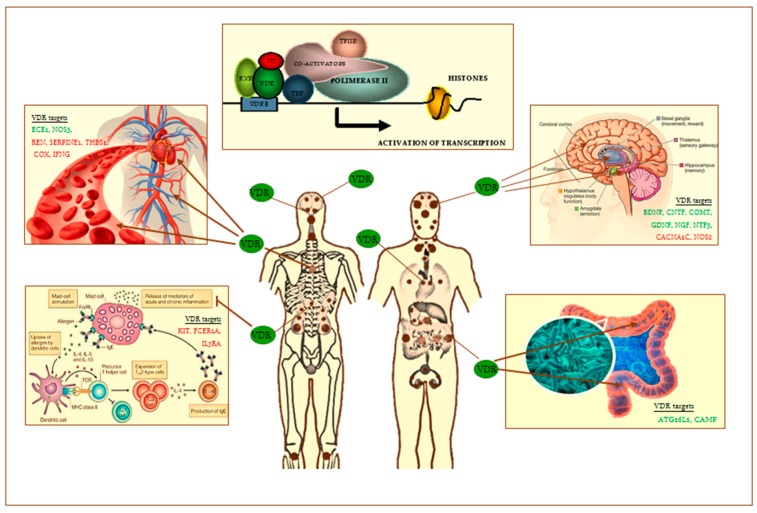
VDR heterodimer transcription complex and VDR distribution throughout the human body (brown small and big dots) and. Tissue targets of VDR are the following: skin, brain regions, spinal cord, pituitary gland, salivary glands, nasal-oral mucosa, teeth, parathyroid, thymus, lung, heart, spleen, pancreas, adrenal gland, kidney, esophagus, stomach, pylorus, small intestine, large intestine, testis, prostate, bone, immune cells, others. On the left and right of the figure are shown some of VDR old (cardiovascular system) and emerging tissue targets (brain, intestinal microbioma, mast cells). Some of main known VDR gene targets in these latter are shown (**in green**, up-regulated; **in red**, down-regulated): *ATG16L1*, Autophagy-related protein 16-1; *BDNF*, brain-derived neurotrophic factor; *CACNA1C*, L-type voltage-sensitive calcium channel subunit A1C; *CAMP*, cathelicidin antimicrobial peptide; *CNTF*, ciliary neurotrophic factor; *COMT*, catechol-O-methyl-transferase; *COX*, cycloxygenase; *DRD2*, dopamine receptor D2; *ECE1*, Endothelin 1-converting enzyme; *FCER1A*, Fc fragment of IgE receptor IA; *GDNF*, glia-derived neurotrophic factor; *IFNG*, Interferon-γ; *IL3RA*, IL-3 receptor-alpha chain; *KIT*, gene-encoding CD117; *NGF*, nerve growth factor; *NOS3*, endothelial nitric oxide synthase; *NT3*, neurotrophin 3; Nurr1, nuclear receptor related 1 protein; *SERPINE1*, serine-protease inhibitor 1 (PAI-1); *REN*, Renin; *RXR*, retinoid X receptor; *TBP*, TATA-binding protein; *TFIIB*, transcripton factor IIB; *THBS1*, Thrombospondin-1; *TRPV6*, transient receptor potential cation channel subfamily V member 6; *VDRE*, vitamin D response elements on DNA sequence; *VD*, vitamin D.

Vitamin D and its analogs are natural ligands of a nuclear receptor, the vitamin D receptor (VDR), widely distributed in various cell types, organs and tissues (Figure 1) [10]. Recent epidemiological investigations suggest that the prevention of chronic diseases may be achieved by increasing vitamin D levels in the normal range. Thus, as the dietary intake of vitamin D is often inadequate, supplementation with either vitamin D2 or vitamin D3 or their analogs, at a daily dose and tolerable upper-limit levels, should be considered. However, despite demonstrated health benefits coming from vitamin D supplementation [10], evidence on how vitamin D might function in the case of the aforementioned diseases, except osteoporosis and rickets, is still lacking.

## 3. Vitamin D Genomic, Epigenomic and Non-Genomic Actions

The genomic actions of vitamin D are dependent on its ability to regulate gene transcription through the binding of VDR to the retinoid receptor (RXR). VDR/RXR is a heterodimer complex able to bind to specific vitamin D response elements (VDRE), present in several gene promoters, and either activate or inhibit the transcription of genetic targets involved in various cellular processes (Figure 1) [10]. The VDR/RXR heterodimer also interacts with co-regulatory factors, i.e., the steroid receptor co-activator family, the CREB-binding protein/p300, and remodeling factors that influence chromatin architecture in a gene-specific way by affecting the epigenetic status and even the gene locus overall organization [11]. It has also been shown that vitamin D-activated gene transcription may be regulated from distal sites at gene promoters by multiple enhancers that are interspersed in intronic and intergenic regions [12]. Notably, VDR has also been shown to work independently from vitamin D binding, even if it is likely that only a few genes are directly regulated by this way [12].

The VDR/RXR complex is involved in the regulation of many key transcription pathways, the most important of which are those activated by insulin, transforming the growth factor beta1, Wnt and various cytokines [13]. Vitamin D is also able to modulate important cellular processes, such as autophagy, apoptosis, cell cycle, endocytosis, cell adhesion, axon guidance, and actin remodeling [14]. Hence, the regulation of transcriptional processes by the activated VDR/RXR complex is responsible for phenotypic stability.

The same VDR/RXR complex is able to regulate epigenetic mechanisms involved in the maintenance of transcription processes. An example is represented by VDR/RXR-mediated recruitement of histone acetyltransferases that promote acetylation reactions causing chromatin deconcentration and facilitating gene transcription [15]. Vitamin D is also able to regulate the methylation status of CpG islands in the promoters of its gene targets by inducing the expression of key histone demethylases, namely lysine-specific demethylases 1 and 2 (LSD1, LSD2) and JmjC domain-containing 3 (JMJD3) [15]. Hypermethylation of gene promoters increases with aging and may be involved in the onset of several age-related disorders, such as cancer, cardiovascular, and neurological diseases, that have been associated with low plasma levels of vitamin D [15].

Vitamin D non-genomic actions are related to the activation of VDR, in a different configuration, and ERp57/GRp58/ERp60, also known as MARRS (membrane-associated rapid response steroid-binding) protein, both located within caveolae/lipid rafts in the membrane. The stimulation of these receptors is linked to the activation of signalling pathways involving kinases, phosphatases, and ion channels [16].

## 4. Regulation of Redox/Detoxification Metabolism by Vitamin D

Normal cell functions and survival are guaranteed by the maintenance of a highly reduced internal environment of cells. A shift towards an oxidized state, caused by the increase in the levels of intracellular reactive oxygen species (ROS), leads to redox stress-induced impairment of cell homeostasis [17]. ROS formation occurs at two main sites, the mitochondria and the cell membrane. In mitochondria, ROS are generated as a consequence of transmembrane mitochondrial potential disruption induced by toxic stimuli [18]. The stimulation of G protein-coupled receptors (GPCR) and receptor tyrosine kinases (RTKs) on the cell membrane by growth factors, hormones and cytokines, leads to the activation of their downstream effector phosphatidyl-inositol-3-kinase (PI 3-K). Activated PI-3K produces the second messenger phosphatidyl-inositol-3,4,5-phosphate (PIP3), stimulating NADPH oxidase (NOX) to generate the anion superoxide (O_2_^−**.**^), which is transformed to hydrogen peroxide (H_2_O_2_) by superoxide dismutase (SOD). NOX may be also activated by the receptor for advanced glycation-end products (RAGE), the expression of which is modulated by vitamin D [19]. RAGE-induced inflammation is observed in several pathological conditions associated with vitamin D deficiency, i.e., diabetes, cancer, cardiovascular disorders, and neurodegenerative diseases [20].

Cells have evolved sophisticated defenses against oxidative stress that are represented by detoxifying and antioxidant enzymes able to remove ROS and also to reverse the oxidative changes occurring upon the physiological production of ROS needed for intracellular signaling [21]. Vitamin D is able to regulate the expression of many antioxidant systems. As an example, vitamin D performs a control action on the expression of the nuclear factor-erythroid-2-related factor 2 (Nrf2), a redox-sensitive transcription factor able to activate numerous genes encoding for antioxidant and detoxifying enzymes [19]. The transcriptional activity of Nrf2 is usually inhibited through its binding with Keap 1, acting as the adapter protein for Cullin 3, an ubiquitin ligase driving the degradation of Nrf2 via the ubiquitine/proteasome pathway. However, when ROS levels increase, Nrf2 is released upon Keap 1 oxidation, enters the nucleus and promotes gene transcription through the binding to antioxidant response elements (ARE) present in the promoter region of genes encoding for detoxifying and antioxidant enzymes, such as cytochrome P450, glutathione S-transferase, sulfotransferase, glutamate cysteine ligase, heme oxigenase 1, superoxide dismutase 1 and 2 (SOD1, SOD2), catalase, glutathione peroxidase , NAD(P)H quinone oxidase 1, peroxyredoxins (Prxs), and thioredoxin (Trx). Notably, Nrf2 is able to induce the up-regulation of VDR and RXR, through increased expression of Fos and Jun, in order to enhance cell sensitivity to low vitamin D levels [22].

Vitamin D is also able to regulate the expression of Klotho, a trans-membrane protein having a small cytosolic domain and a large extracellular domain, which is cleaved by ADAM 10 and ADAM17 and released as a humoral factor to influence several signaling pathways and cellular processes [23]. Klotho displays an antioxidant action through two main pathways: (1) inhibition of insulin/IGF1, resulting in FoxO activation that, in turn, induces the up-regulation of mitochondrial SOD2 and catalase [24]; (2) induced phosphorylation of the PI3K/Akt pathway, resulting in the increased expression of peroxiredoxins and thioredoxin reductase 1 [25].

Vitamin D deficiency likely leads to a reduced expression of Klotho and Nrf2 and, as a consequence, to the disruption of redox system homeostasis. The loss of this control is determinant for the onset of numerous age-related disorders.

## 5. Vitamin D, its Analogs and Cardiovascular Disorders

In the last decade, evidence has been provided for the association of low vitamin D levels with increased risk of hypertension, CHD, and stroke. However, there is still conflicting information on the effective preventive action of vitamin D against cardiovascular diseases (CVD) [26]. Systematic reviews carried out in United States (US) highlighted that evidence available was inconclusive and not strong enough to establish a cause–effect relationship [27]. Numerous studies showed that vitamin D plasma concentration in a range 20–30 ng /mL is inversely correlated with the incidence of cardiovascular adverse events [4,28,29]. In the NHANES III database, although the association between vitamin D and mortality for CVD was found not to be significant, an increased mortality rate in subjects having vitamin D values below 20 ng/mL and above 50 ng/mL was observed [28]. Moreover, evidence has been provided for the correlation between vitamin D values below 30 ng/mL and arterial hypertension [30,31,27].

VDR is widely distributed throughout the vascular system. Notably, the enzyme 1α-hydroxylase that catalyses 25-hydroxyvitamin D activation to calcitriol, the natural ligand of VDR, is expressed on endothelial cells, vascular smooth-muscle cells, and cardiomyocytes. VDR activation in endothelial cells influences angiogenesis, acting on response elements in the promoter region of the vascular endothelial growth factor (*VEGF*) gene [32]. Calcitriol preserves vascular system functions through several mechanisms: (1) inhibition of vascular smooth-muscle cells proliferation, intima-media thickening and metalloproteinase activity; (2) enhancement of vasodilatory action of endothelial nitric oxide synthase; (3) prevention of the development of atherosclerotic plaques (inhibition of macrophages conversion into foam cells); (4) prevention of calcium deposition in vessels; (5) endothelium protection against advanced glycation end products (AGEs); and (6) anti-thrombotic activity [32,33,34,35,36]. Some of these effects take place through a genomic action of VDR (Figure 1).

Moreover, evidence provided from studies on animals and humans suggests that vitamin D prevent hypertension by mediating a negative feedback on the renin–angiotensin–aldosterone (RAA) axis, responsible for angiotensin II-mediated vascular resistance and the maintenance of extracellular fluid volume homeostasis. Vitamin D reduces RAA activity through a *cis*-mediated down-regulation of renin gene expression [37]. In VDR knockout mice, there is a higher rate of hypertension, water retention and cardiomyopathy due to ventricular dilatation and impaired electromechanical coupling, caused by left ventricle hypertrophy, increased concentration of atrial natriuretic peptides, and the formation of fibrotic extracellular matrix generated by metalloproteinases and fibroblasts’ homeostasis impairment [38,39]. In vitro vitamin D has been observed to reduce both the entry of calcium ions into cardiac muscle cells and the deposition of collagen, and to remodulate both myosin chain distribution and cardiomyocyte differentiation [40,41].

It has been shown that the chronic treatment of spontaneously hypertensive rats with vitamin D reduces hypertension and contractions of aortic endothelial cells via modulation of several protein kinase pathways [42]. A clinical trial showed that arterial blood pressure may be lowered by vitamin D administration leading to reduced circulating concentrations of renin, angiotensin and aldosterone [43].

Chronic heart failure is also associated with increased levels of circulating pro-inflammatory cytokines, i.e., tumor necrosis factor-α (TNF-α), interleukin-1 (IL-1), and interleukin-6 (IL-6). Vitamin D supplementation in patients with heart failure has been shown to display specific, but modest anti-inflammatory effects [44].

The use of calcitriol in the treatment of cardiovascular disorders is often associated with the appearance of dose-dependent adverse effects, such as hypercalcaemia, hypercalciuria, and the formation of calcifications in the parenchyma of various organs [26]. In light of this, new calcitriol analogs have been synthesized which have a reduced or absent activity on calcium metabolism. There are several calcitriol analogs with different biological activities that are currently used in clinical and preclinical trials. The major concerns about the use of calcitriol analogs are their affinity for VDR, metabolism by the cytochrome P450, and transport by DBP and other proteins [8]. Paricalcitol and maxacalcitol are selective VDR activators (VDRAs), and are able to reduce calcium absorption and potassium elimination, and to activate selective metabolic pathways [9].

There is scientific evidence demonstrating the efficacy of VDRAs to improve ventricular function in both human and experimental models [45]. The use of VDRA resulted in an improvement in left ventricular function and a reduction of left ventricular hypertrophy in cardiopathic patients [45,46]. VDRAs have been shown to reduce myocardial fibrosis in mouse models, and these effects may be mediated by miRNAs [47,48]. Notably, paricalcitol have a therapeutic advantage compared to calcitriol that is consistent with the suppression of renin production (reduced side effects of hypercalcemia) and lack of vascular calcium deposition, which instead causes aortic calcifications with arterial stiffness and hypertension regardless of plasma Ca and P levels. The reason for the selective effect of paricalcitol has to be better elucidated since its clearance from the bloodstream is comparable to that of 1,25(OH)2D [47,48,49,50,51].

## 6. Vitamin D and Neurological Disorders

In the last decade a large body of literature data from epidemiological studies, animal experiments and clinical trials showed the link between vitamin D deficiency and/or VDR gene polymorphisms and several central or peripheral neurodegenerative diseases, particularly multiple sclerosis (MS), amyotrophic lateral sclerosis, Parkinson’s disease (PD) and Alzheimer’s disease (AD), and neuropsychiatric disorders, i.e., depression, autism and schizophrenia [52].

In the central nervous system (CNS), vitamin D is synthesised and locally metabolised due to the expression of CYP27B1 in neuronal cells and glia. VDR is abundantly present in various brain regions, particularly in the cerebellum, hippocampus, substantia nigra, basal forebrain, prefrontal cortex and hypothalamus [53]. This may explain the role of vitamin D in the regulation of pivotal processes both in developing and adult brains, through genomic and non-genomic actions. Vitamin D genomic actions in developing brains involve the regulation of neural stem cell proliferation and differentiation, oligodendrocyte differentiation, and dopaminergic neurons development, while in the adult brain they modulate neurogenesis in the hippocampal region and the synthesis/release of neurotransmitters, such as dopamine, γ-aminobutyric acid (GABA), and serotonin [54]. In the aged brain, vitamin D genomic actions are aimed at neuroprotection through the release of neurotrophic factors, the prevention of excitotoxic damage and blood–brain barrier (BBB) disruption, the synthesis of neurosteroids in glial cells, and the attenuation of inflammatory response through immunomodulation [54]. Accordingly, available data from murine experimental models showed that the exposure to vitamin D deficiency in early life causes changes in behavior and alterations of brain structures as well as dopaminergic neurotransmission, while in adult life behavioral and neurochemical changes are more subtle [55]. Non-genomic actions of vitamin D in the brain are linked to the modulation of synaptic transmission and intracellular signaling activated by calcium and kinases [54]. In light of these findings, a question arises about the existence of a critical window during which exposure to vitamin D deficiency may increase the risk of neurological impairment. A “two-hit” hypothesis has been proposed suggesting that an early exposure to low vitamin D levels makes the brain more susceptible to subsequent exposures, increasing the risk for the onset and progression of neurodegenerative diseases [55].

One of the most relevant neuroprotective mechanisms activated by vitamin D and its metabolites is the down-regulation of the l-type voltage-sensitive calcium channel (LVCC), the activity of which induces excitotoxic cell death in the hippocampus of the aged brain [56]. Calcitriol-induced up-regulation of VDR has also been shown to attenuate apoptotic cell death induced by autophagy dysfunction [57]. Vitamin D3 also protects the nervous system against oxidative stress-induced apoptosis through VDR-mediated down-regulation of NOX [58] and inducible nitric oxide synthase (iNOS) (Figure 1) [54], an enzyme which usually produces nitric oxide (NO) at a high-output rate in an oxidative environment. In these redox conditions, high NO levels may easily react with the superoxide anion leading to the formation of peroxynitrite, a highly reactive free radical, and cell toxicity [54]. Moreover, vitamin D3 stimulates γ-glutamyl transpeptidase activity that is required for the synthesis of glutathione, a non-enzymatic, key antioxidant defense within cells [54].

Vitamin D3 also modulates nerve growth by inducing the production of neurotrophic factors, i.e., glial cell-derived neurotrophic factor (GDNF), nerve-growth factor (NGF), and neurotrophin 3 (NT-3) [59] (Figure 1). Moreover, vitamin D and the VDRA calcipotriol have been shown to increase the expression of aromatase, an enzyme capable of converting androgens into estrogen, thus increasing local estrogen endogenous production in glial cells that, in turn, ensures the maintenance of neuronal functions [59,60].

In addition, vitamin D plays a role in synaptic plasticity, as suggested by its inhibitory action on Na^+^ and Ca^+^ currents stimulated by kainate and NMDA within neuronal cells, as well as a blocking action on the frequency of GABAergic postsynaptic currents [61], and its ability to restore extracellular postsynaptic potentials in CA3 and CA1 regions of the hippocampus [62].

The classic VDR nuclear receptor is localized with caveolin-1 in the membrane of neurons as well as in other types of cells [63]. Studies based on co-immunoprecipitation reveal that VDR and protein disulfide-isomerase A3 (PDIA3) interact with non-nuclear compartments. Indeed, siRNA antibodies against PDIA3 transcripts are capable of blocking and inhibiting rapid non-genomic responses mediated by vitamin D [64,65]. Moreover, the action of calcium ion as the second messenger remains extracellular until it enters the brain due to vitamin D action, which allows the activation of several calcium-dependent signalling pathways such as those of PKA, PKCaMII, p38MAPK and PI3K in many cellular lines [66].

### 6.1. Vitamin D, Cognitive Impairment, Vascular Dementia and AD

Notably, vitamin D levels have been shown to be inversely correlated with the risk of developing vascular calcification [67], a well-known marker of atherosclerosis and a risk factor for dementia development [68]. In the elderly, severe vitamin D deficiency has been associated with cognitive impairment, dementia and the development of AD [69,70]. Two meta-analysis studies, including only cross-sectional and longitudinal, but not prospective, investigations, indicated that increasing vitamin D levels were associated with higher average scores in minimal mental state examination (MMSE) tests, while low vitamin D levels increased by 2.4-fold the risk for cognitive decline and dementia [71,72]. Vitamin D deficiency has been linked to smaller volumes in AD-related brain regions, reduced cognitive function, and reduced cognitive performance in the domains of memory and executive functions [73,74]. Vitamin D supplementation may exert a protective action [75]. AD has been associated with VDR *Taq*I and *Apa*I gene polymorphisms [76,77], which further suggest a role for vitamin D in the onset of neurocognitive impairment. 

Vitamin D deficiency may be involved in AD development because of the lack of vitamin D-driven protective effects related to the enhancement of clearance and phagocytosis of amyloid-beta (Aβ) peptide across the blood–brain barrier [78], and the prevention of cortical neurons degeneration induced by Aβ through LVCC down-regulation mediated by VDR up-regulation [56,79].

Studies suggest that vitamin D can increase the levels of P-glycoprotein (P-gp), a membrane protein that pumps numerous dangerous molecules out of the brain, in the endothelium of capillary cells of humans and rats [80], as well as the expression of low-density lipoprotein 1 (LPR-1), a protein having the ability to increase clusters of Aβ [81].

Vitamin D has been shown to crosstalk with estrogen and insulin signaling to indirectly regulate molecular pathways relevant to AD, such as the immune/inflammatory response, neurotransmission, vascular processes, and hormonal alterations [82].

### 6.2. Vitamin D, Dopaminergic Signaling and PD

Immunohistochemical studies revealed the presence of VDR in rodent and human substantia nigra, suggesting that the dopaminergic system is modulated by vitamin D [83]. Vitamin D has been shown to reduce the death of dopaminergic neurons in PD mice and increase local dopamine production [84]. Vitamin D supplementation has been introduced in numerous clinical trials in PD patients due to its ability to stimulate VDR expression in substantia nigra. Several lines of evidence indicate that vitamin D can positively modulate cerebral functions in the healthy subject, and even to a greater extent in PD subjects [85].

Vitamin D deficiency decreases the expression of tyrosine hydroxylase (TH) and nuclear receptor-related 1 protein (Nurr1), which plays a key role in the maintenance of the dopaminergic system of the brain, in the rodent hippocampus [86]. Alterations in the expression of Nurr1 during the window of dopaminergic development could affect the distribution and development of dopaminergic neurons. Such disequilibrium occurs with dopamine-related adverse behaviors in adult offspring such as motor spasticity [87]. These alterations are due to changes in the dopamine transporter (DAT) in striatum and nucleus accumbens; in fact, vitamin D deficiency alters the density and distribution of DAT in the two aforementioned brain areas. Nurr1 is responsible for these changes, since it controls the TH and DAT pathways essential for dopaminergic system development [87].

Vitamin D also controls dopaminergic metabolism through a genomic action on the promoter of the catechol-*O*-methyl-transferase (*COMT*) gene which is an enzyme responsible for dopamine methylation. Vitamin D administration results in the increased expression of COMT in VDR-expressing SH-SY5Y cells and neuronal cells, and in the regulation of many genes responsible for the development and maintenance of dopaminergic neurons, such as those encoding for neurogenin 2 (*NEUROG2*), dopamine receptor 2 (*DRD2*), monoamine oxidase A (*MAO-A*) and vesicular monoamine transporter (*VMAT2*) [87] (Figure 1).

Vitamin D increases GDNF levels in glia and primary neurons, as well as in the striatum of both healthy and Parkinson’s disease rodent models; notably, GDNF depletion occurs in the brain of vitamin D-deficient rats [88].

A recent meta-analysis has shown that VDR *Bsm*I gene polymorphism is associated with PD in the Asian population [77], while *Fok*I gene polymorphism is related to PD in the general population [89].

Calcipotriol was shown to exert a neuroprotective action through the activation of calbindin-D-28k, which is able to block the calcium-mediated depolarization depending on the aggregation of α-synuclein [90]. This study represents the meeting point between genomic and genomic actions of vitamin D, suggesting that vitamin D can modulate and change the excitation and inhibition pathways of local neuronal circuits.

### 6.3. Vitamin D and Neuroinflammation

Neuroinflammation is one of the mechanisms responsible for memory loss and cognitive decline in adults. In 20-month mice, vitamin D injection for 21 days has been shown to improve cognitive function and cognitive-related impairment through the reduction of pro-inflammatory IL-1β levels and increase anti-inflammatory IL-10 levels in adult mice [91]. Then, the increase in IL-10 would inhibit the synthesis of many pro-inflammatory cytokines, which may act on CNS as triggers of neuroinflammation, through a pathway mediated by the suppressor of cytokine signaling-3 (SOCS3) [92]. The anti-inflammatory action of vitamin D has been shown in vitro with lipopolysaccharide (LPS) or interferon a-activated glia. In fact, the administration of vitamin D in LPS-stimulated microglia results in a decrease in IL-2, IL-12 and TNF-α expression induced by LPS [92].

Vitamin D supplementation influences brain immunomodulation. In preclinical studies under the action of LPS or poly (I:C) (polyinosinic-polycytidylic acid), microglia isolated from vitamin D-deficient mice produce less IL-6 and TNF-α, and there is also a lower phagocytic action and intracellular killing compared to mice with sufficient amounts of vitamin D [93].

By the use of the active experimental autoimmune encephalomyelitis (EAE) rodent model of MS, it has been shown that myelin injection in animals activates an immune response leading to CNS inflammation and paresis with symptoms similar to MS. Incidence, severity and course were ameliorated by vitamin D supplementation, even if only in female mice [94]. This may be explained by a synergy occurring between estrogens and vitamin D that involves an estradiol-mediated up-regulation of VDR expression in the inflamed brain and a vitamin D-activated increase of estradiol synthesis [94].

Recent work showed that the transfer of vitamin D-induced tolerogenic dendritic cells was able to suppress EAE through the increase of regulatory B and T cells, as well as CD4^+^ IL-10^+^ T cells in mouse spleen, and the reduction of infiltrated Th1 and Th17 cells into mouse spinal cord [95]. Moreover, vitamin D treatment significantly attenuated inflammatory processes through the reduction of serum and spinal cord concentrations of IFN-γ, IL-17A, IL-23 P19, IL-23 P40, GM-CSF pro-inflammatory cytokines, as well as CCL20, CCL22 and CCR4chemokines, related to TH17 lymphocyte activity, and the increase of IL-4 and IL-10anti-inflammatory cytokines. Furthermore, 1,25(OH)2D3 treatment effectively elevated the numbers of neural stem cells, oligodendrocyte precursor cells, as well as oligodendrocytes in disease lesions in the CNS [96,97].

It has been found that calcitriol supplementation stimulates myelination and remyelination in mouse models with neuronal damage and multiple sclerosis. This action is also immunomodulated and probably mediated by the microglia that is known to be imported for the remyelination process [98].

### 6.4. Vitamin D and Stroke

High levels of C reactive protein (CRP) have been found in vitamin D-deficient subjects with acute ischemic stroke. Negative feedback between increased levels of vitamin d and low CRP levels explains the anti-inflammatory function of vitamin D during acute ischemic stroke [99].

It has also been shown that vitamin D prevents hypoxia/re-oxygenation occurring after the destruction of BBB because of NF-κB activation [100]. Moreover, intranasal administration of vitamin D protects against subaracnoid haemorrhagic stroke induced by the destruction of BBB through the up-regulation of P-gp in endothelial cells [101].

## 7. Vitamin D and Immune System

### 7.1. Hypovitaminos D and Mast Cell Activation

Epidemiological studies show that vitamin D deficiency increases the individual susceptibility to pathogen infections. Hence, a new role for vitamin D3 as immunomodulator has been claimed and vitamin D supplementation has been suggested as a preventive strategy of some infectious diseases [102]. Indeed, in the immune cell system VDR is expressed by neutrophils, CD4^+^ and CD8^+^ T lymphocytes, antigen-presenting cells (dendritic cells, macrophages) and B lymphocytes, particularly when they are stimulated by pathogens. Notably, these cells are able to activate 25(OH)vitamin D3 to 1,25-(OH)_2_vitamin D3 due to the presence of CYP27B1. VDR activation may lead to the inhibition of transcription of cytokine-encoding genes [103]. Moreover, calcitriol can block IgE-mediated mast cell degranulation (Figure 1).

Mast cells are the major effector cells in allergic disorders and many other inflammatory disorders. The mechanism of mast-cell stabilization is not fully understood. A recent investigation by Liu and co-workers [104] demonstrated that vitamin D3 is required in the maintenance of mast cells’ stability. The experimental design involved the incubation of different cultured mast cells, deriving from RBL-2H3, p815, HMC1 cell lines or mouse bone marrow (BMMC) with or without calcitriol for three days. Mast cells cultured without vitamin D were found to be automatically activated, as indicated by an approximately 15–30% higher content of histamine and TNF-α in the cell-culture supernatant. These mediators were released from vital mast cells (BMMC 98.4%, HMC1 98.8%, RBL-2H3 99.1%, p815 98.2%). Exposure to calcitriol increased the expression of VDR in mast cells. VDR was able to bind the tyrosine kinase Lyn, that is usually responsible for the immediate activation of FcεRI after the interation of this latter with multivalent antigens or after exposure to the lipopolysaccharide microbial product. The complex VDR/Lyn prevented the binding of Lyn to the β chain of FcεRI and MyD88, which are the two signalling pathways of greater mast-cell activation. This resulted in a reduced phosphorylation of Syk, a cytosolic protein kinase responsible for the phosphorylation/activation of linker of activated T cells (LAT) which represents a mediator of mast cells’ degranulation, and decreased levels of MAPK p38 and NF-κB. Moreover, VDR binding to TNF-α promoter was able to decrease the acetylation at the promoter region of histone H3/H4, RNA polymerase II and OCT1 (a transcription factor of TNF-α), and induce the down-regulation of TNF-α in mast cells [104]. These findings demonstrated that in a vitamin D-free environment mast cells are able to release inflammatory mediators even in the absence of activating factors. On the contrary, vitamin D3 is able to maintain the stability of mast cells because of VDR interaction with cytosolic molecules modulating the mast-cell activation state.

The activation of mast-cell degranulation is responsible for the pathogenesis of many inflammatory immune diseases [105]. Although many mast-cell antagonists have been identified, it has been observed that they have a short-lived inhibitory action on mast-cell activation [106]. The presence of vitamin D was able to inhibit mast-cell activation induced by IgE-mediated sensitization. Notably, an inadequate or deficient plasma vitamin D concentration can be one of the causal factors of VDR insufficiency or deficiency in mast cells. VDR is implicated in both nuclear and cytoplasmic biochemical reactions. In the latter case, it is involved in various biosynthetic pathways such as the tricarboxylic acids and the acetyl-CoA-dependent pathways [107]. Numerous inflammatory mediators produced by activated mast cells are regulated by vitamin D; however, adjustments on many of them still need to be studied.

Animal studies demonstrating the benefit of vitamin D3 and its analogs in the treatment of autoimmune diseases [108], and as adjunct immunosuppressants following transplantation procedures [109], are also compelling, but as for the treatment of infections randomized clinical trial data are lacking.

### 7.2. Hypovitaminosis D and Hematological Malignancies

Since VDR is expressed in peripheral blood mononucleocytes (PBMCs), several investigations have addressed whether vitamin D plays a role in haematological disorders, particularly focusing on blood cancers. Vitamin D deficiency has been associated with hematological malignancies, including leukemia, lymphoma and myeloma, and with a poor prognosis for these diseases [110]. Despite advances in the therapeutic management of lymphatic neoplasias, no significant improvement of overall survival has been achieved so far.

Macrophage infiltration is a pathognomonic sign of high-grade lymphatic malignancies. However, it has been observed that macrophage-mediated antibody-dependent cytotoxicity regularly fail in these disorders for still unexplained reasons. Recent work demonstrated that vitamin D is able to stimulate an effective cytotoxic action of inflammatory M1 macrophages against proliferating high-grade B cell lymphoma cells through the release of cathelicidin, an antimicrobial peptide. Cathelicidin-mediated cell death occur by mitochondria-targeting in tumor cells. Notably, the treatment of anti-inflammatory M2 macrophages and M2-like tumor associated macrophages with 1,25 (OH)2 vitamin D3 or synthetic analogs, makes them able to synthetize and release cathelicidin, eventually killing lymphoma cells. The supplementation of vitamin D in vitamin D-deficient lymphoma patients improves the efficacy of rituximab cytotoxic activity, which is cathelicidin-dependent [111].

The treatment with either vitamin D3 or its analogs has been shown to be effective against acute myeloid leukemia, and has recently been regarded as a potential therapeutic approach for other hematological tumors. In particular, experimental studies have shown that VDAs display cytotoxic effects in a time- and concentration-dependent manner on large B-cells’ lymphoma and healthy B-cells [112]. Moreover, vitamin D and VDAs have been shown to induce proliferation inhibition and onset of differentiation, apoptosis, reduction of pro-inflammatory cytokine release, and tumor-cell sensitization to other anti-cancer therapies in blood tumor cells [113].

## 8. Vitamin D and the Gastrointestinal Microbioma

Low vitamin D levels have been found in patients with gastrointestinal disorders (GID), i.e., Chron’s disease, inflammatory bowel disease, and colitis [114]. Clinical trials showed that the quality of life of GID patients can be improved through supplementation with recommended doses of vitamin D [115]. Experimental studies have shown that low vitamin D levels lead to the disruption of tight junctions’ integrity and the development of intestinal barrier dysfunction, mucosal damage, and increased susceptibility to infections [116,117,118]. These data highlight that vitamin D and VDR are required to maintain gut homeostasis and protect the intestine from injuries, goals that are likely achieved through the control of the innate immune response of the microbioma, microbial dysbiosis, and maintenance of intestinal immune tolerance [119].

Oral supplementation of vitamin D3 causes an alteration in the composition of gastrointestinal microbioma, characterized by a reduction in opportunistic pathogens and an increase in bacterial variability [120].

Recent studies demonstrated that VDR status modulates the composition and functions of intestinal bacterial population. VDR^−/−^ mice are more prone than wild-type ones to develop intestinal dysbiosis- characterized by a depletion of *Lactobacillus* and butyrate-producing bacteria, and an increase in *E. coli*, *Clostridium* and *Bacteroides-* and severe colitis induced by dextran sodium sulfate (DSS)-mediated chemical insult [121]. Intestinal epithelial VDR deletion leads to defective autophagy, through the regulation of *ATG16L1* gene expression in experimentally-induced colitis [121] (Figure 1). Notably, a genome-wide host-microbiota association analysis of 1,812 individuals showed that VDR gene variation and other host factors shape the gut microbiota, and there are correlations between the microbiota and serum levels of bile and fatty acids [122]. 

An alternative approach to prevent and treat intestinal inflammatory disorders is the administration of probiotics, such as *Lactobacillus*, *Bifidobacterium*, and *Saccharomyces*, that are able to maintain/restore intestinal homeostasis through the balancing of commensal and pathogenic bacteria, attenuation of symptoms, regulation of innate immune system through the control of TLRs, NF-κB, MAPK pro-inflammatory pathways, modulation of intestinal metabolism, and modification of toxic compounds and host products [123,124].

Evidence has been provided that probiotic treatment can increase vitamin D levels as well as VDR expression and transcriptional activity in the host, and that VDR plays a key role in the protective effects of probiotics against inflammation and infection [125,126].

*Lactobacilli* are able to increase the expression of VDR target-gene encoding for cathelicidin (Figure 1), a cationic antimicrobial peptide, and to protect wild-type mice from *Salmonella*-induced colitis, while failing to inhibit *Salmonella* infective action in VDR^−/−^ mice [124].

The restoration of VDR expression on the surface of inflammatory intestinal mucosa through the administration of selected probiotics strains would represent a treatment strategy for IBDs and other inflammatory diseases.

## 9. Conclusions and Perspectives

In recent years, thousands of VDRE sites on the DNA sequence have been identified. Given the almost ubiquitous expression of VDR and CYP27B1, a great effort still has to be made to characterize molecular pathways regulated through genomic and non-genomic actions of this vitamin. Moreover, it is also very important to look for strategies to target specific cells with vitamin D analogs that do not display adverse side effects, such as increased intestinal calcium absorption and/or bone resorption. At present, analogs have been developed only for the therapeutic management of osteoporosis, hyperparathyroidism, and skin hyperproliferative disorders [10]. However, there is still a great demand for solid data from randomized clinical trials aimed at the treatment/prevention of cancer, CVD, neurodegenerative disorders, infections, and autoimmune diseases.

Findings from genome-wide analyses suggest the occurrence of several variants of key proteins of vitamin D metabolism that may affect circulating concentrations of vitamin D metabolites. These proteins include VDR, DBP, and 7-dehydrocholesterol synthase [6].

It is reasonable to expect in the near future that these protein variants will be in vivo detected and the above cited issues will be overcome, so that the management of disorders developing in association with vitamin D deficiency will be greatly improved.
